# Future Perspectives of Finite-Time Thermodynamics

**DOI:** 10.3390/e24050690

**Published:** 2022-05-13

**Authors:** Bjarne Andresen, Peter Salamon

**Affiliations:** 1 Niels Bohr Institute, University of Copenhagen, Blegdamsvej 17, DK-2100 Copenhagen Ø, Denmark; 2 Department of Mathematics and Statistics, San Diego State University, 5500 Campanile Drive, San Diego, CA 92182-7720, USA

Finite-time thermodynamics was created 45 years ago as a slight modification of classical thermodynamics, by adding the constraint that the process in question goes to completion within a finite length of time. It was started using very simple models of real processes and has since then evolved into many new areas as clearly evidenced by the 20 quite diverse papers of this Special Issue “Finite-Time Thermodynamics” [1,2,3,4,5,6,7,8,9,10,11,12,13,14,15,16,17,18,19,20]. The philosophy has remained the same: add a time constraint for the processes involved and optimize for your desired quantity (power, efficiency, profit, population, whatever). But the types of processes considered and the accuracy of the modeling have expanded immensely from the original simple Curzon-Ahlborn engine.

While much work is still needed optimizing concrete and very important processes and machines in our daily lives, we would here like to raise our gaze to more distant horizons. Where could the field go next to expand our insight and modeling abilities?

Some of the most promising directions we are imagining are beyond what is traditionally considered thermodynamics. One direction which contains many astonishing concepts, and which has already been underway for many years, is quantum finite-time thermodynamics. Not only will one encounter the ‘usual’ quantum effects, some very untraditional ones also appear. Quantum resources like coherence and entanglement carry available work. It follows that Carnot engines working between heat reservoirs containing such “hidden” resources can appear to violate Carnot’s bound [21]. Similarly, systems with bounded upper energy levels can exhibit negative temperatures. Such systems can turn a heat input into pure work without further flows. Recent “shortcuts to equilibrium” [22] can take a system from one equilibrium state to another equilibrium state within a short period of time as opposed to a usual adiabatic transformation which in principle would require an infinite length of time. In other words, we can generate reversible transformations in lossy systems taking place in a finite time by appropriate controls. As quantum computing using q-bits develop, such *quantum finite-time thermodynamic considerations in lossy environments* and related optimal solutions for design of the physical equipment will become very important to gain the full potential of quantum computing.

One area of particular concern in connection with quantum systems is that they are governed by Hamiltonian dynamics and thus in principle are lossless. By contrast, finite-time thermodynamics always involves losses. Its goal is to minimize these losses and calculate the optimal paths using available controls, not just natural free flows. Thus what we need to develop is a *general thermodynamic description of open quantum systems*, i.e., including the lossy interaction with external reservoirs [23,24]. The Lindblad superoperator has been extensively used for this purpose, but is it general enough for quantum computing, and how can we control the rate of transfers, the core component of finite-time thermodynamics, in the loss minimization optimization?

Quantum thermodynamics typically involves translating thermodynamics many orders of magnitude down from the human scale to the atomic scale where many concepts are different (e.g., temperature and pressure). What happens in the opposite direction, moving many orders of magnitude up from the human scale to the galactic scale? A scale typically involves both a spatial and a temporal dimension, often connected by the speed of light. The issue here is that any measurement can be split into 3 regions of accessibility. If we are talking about timescales, there will be a central region which we can observe properly. At times shorter than that, our instruments cannot tell events apart and thus establish “before” and “after” and causality. Processes on timescales longer than our window of observability are not detected since we see no change. Exploring this ‘*slow-time thermodynamics*’ we have just scratched the surface [25] in an attempt to find out which thermodynamic concepts survive (e.g., mass), which ones either disappear or take on a new appearance (e.g., temperature and entropy), and which new ones may emerge. Entropy production, which is a crucial quantity for rate processes, surely will acquire new components from processes which we on human time and space scales can follow in detail but which on the grander scale become impossible to tell apart and thus appear statistical. Wind is such an example. This is a wide open field, not least identifying rate processes, their entropy production rates, and how possibly to control them.

Another virgin territory of finite-time thermodynamics may be found within biology, ranging from the chemical processes of sub-components of single cells (e.g., energy transfer processes in mitochondria) to ecological competition (e.g., corals vs. algae) to evolution. Many attempts have been made at using entropies in comparing the evolution and thus competition of species, ranging from the well established Shannon and Kullback-Leibler forms to home-cooked expressions. While the questions attacked are indeed of great importance for our understanding of biological systems, there is a serious need for a *precise thermodynamic formulation of the basic interactions and objectives of the biological system components* before one can hope for reliable dynamic (finite-time) conclusions. Attempts have been made to model evolutionary steps as phase transitions in the spin glass description of aging. Both are strongly out-of-equilibrium processes with memory and are conceptually similar.

After many years of dormancy, methods based on *thermodynamic geometry* have recently started bearing fruit. The connection between thermodynamic length and dissipation has been used to bound the operation of quantum heat engines for adiabatically driven closed systems [2]. Thermodynamic curvature measures interaction strength, and its use led to better equations of state and shed light on black hole thermodynamics [26]. We anticipate that these are just the beginning and there is much more insights to come from thermodynamic geometry.

*Thermodynamics-like theories* can be used in any area where optimizing behavior is central to the ruling paradigm. These include the disciplines of economics and biology. Optimizing behavior can infuse finite-time thermodynamic ideas and reasoning into such disciplines and leads to carry-over concepts such as dissipation in capital markets [17]. In economics, models assuming optimizing behavior abound with producer optimizing production, consumer optimizing utility, and government optimizing total welfare. These optimization problems give ample opportunity for mapping thermodynamic models into the situation along the lines of mapping mechanics problems into electronic circuits. Much of Georgescu-Roegen’s classic work [27] exploits such mappings. But while it is clear that optimizing behavior is involved, such behaviors in economics and biology stem from the phenomena for which the actual objective function, operating on a given timescale in a given situation, is usually controversial. We expect finite-time thermodynamics to be a future guide to such efforts as a template and as a source of readily adapted models with large palettes of objective functions and associated optimizing behaviors.

Unlike biological or economic systems, computer systems have clear objective functions purely by engineering design. How to achieve optimizing behavior is the main issue here and simulated annealing has been its workhorse example [28]. Optimal cooling depends on the heat capacity and relaxation time of a fictitious physical system that is naturally associated with the optimization problem. Finding similar finite-time thermodynamics based *data mining techniques* to extract the most information with the least work from a database is likely to be a fertile area. This connection to information engines has made great strides [29] and is poised for more.

Standard predictions for the future of engineering involve *designing for sustainability and easy recycling*. But surely the dominant design theme of the next few decades is designing for product intelligence. Since intelligent behavior entails solving some optimization problem under given constraints, our above stated criteria for thermodynamics-like models are fulfilled. Finite time is certainly one of these constraints, so we expect finite-time thermodynamics-like models to play an important role. Since we are approaching the “technological singularity” [30], this may even be the most important development that can benefit from finite-time thermodynamics-like models.
